# Clinical implications of the serum platelet-to-lymphocyte ratio in the modern radiation oncology era: research update and literature review

**DOI:** 10.1186/s13014-024-02485-8

**Published:** 2024-08-13

**Authors:** Dong Soo Lee

**Affiliations:** Department of Radiation Oncology, College of Medicine, The Catholic University of Korea, Seoul, 06591 Republic of Korea

**Keywords:** Biomarker, Cancer, Platelet-to-lymphocyte ratio, Radiation therapy

## Abstract

Radiation therapy (RT) continues to be the primary approach for treating cancer, and numerous cancer biomarkers associated with oncological outcomes have been investigated in the context of RT. The serum platelet-to-lymphocyte ratio (PLR) is one of the emerging landmark biomarker in the oncologic field. Mounting evidence indicates that an elevated serum PLR may function as a marker of unfavorable tumor characteristics, adverse treatment outcomes and treatment-related toxicities among individuals undergoing RT. However, the findings of these investigations have revealed a few disparities among researchers, highlighting the need for further meticulously planned studies to draw conclusive results. This article provides a comprehensive literature review and in-depth discussion regarding the clinical implications of the serum PLR in the modern RT era.

## Introduction

Radiation therapy (RT) has been the cornerstone of numerous cancer treatments [1] since the discovery of its biological efficacy against cancer cells via a variety of mechanisms [2]. Moreover, the recent development of RT technologies has enabled the broad application and supply of RT equipment globally for cancer treatment [3–7].

There are a variety of indices and landmarks available for estimating the benefits and outcomes of RT [8–11]. Blood markers are commonly and regularly tested in cancer patients and have merits in their simplicity, cost-effectiveness, and repeatability [12, 13].

More recently, a number of inflammatory indices based on blood cells have been reported. The lymphocyte-to-monocyte ratio (LMR), neutrophil-to-lymphocyte ratio (NLR), platelet-to- lymphocyte ratio (PLR), and albumin-to-alkaline phosphatase ratio (AAR) are representative examples of widely studied blood inflammatory indices [14–17]. Inflammatory biomarkers are being rigorously investigated because the host immune system and cancer-related inflammation are believed to be linked to the progression and prognosis of a number of malignancies [18]. Furthermore, systemic immune and inflammatory cells, such as lymphocytes, monocytes, neutrophils, and platelets, are thought to play crucial roles in the development of cancer via multiple mechanisms [19, 20].

There is substantial evidence that platelets contribute to cancer development and metastasis [19]. A number of platelet-expressed proteins have been shown to be crucial for tumor spreading in experimental animal models, and platelets have also been implicated in the mechanisms that drive tumor angiogenesis [20–23]. In addition, platelets are considered to protect circulating tumor cells (CTCs) from antitumor immune responses and thereby promote CTC metastasis [12, 24]. Lymphocytes, a well-known type of blood cell, play a vital role in antitumor immune effects and inhibit tumor proliferation and migration [25]. The PLR can therefore serve as one of the primary markers of cancer outcomes.

A number of studies have examined the prognostic relevance of the PLR in different types of cancer [12, 19, 26–30]. However, as multimodal approaches are commonly used in cancer treatment, the role of the PLR in the population of patients who undergo RT has not been well studied. Similarly, during our literature search for this study, we also found that most of the related studies involved patients treated with a variety of management options [31–36]. The objective of this review is to summarize the PLR outcomes in different cancer cohorts of patients who underwent RT.

### Summary of research investigating PLR according to cancer type

We examined the studies related to the PLR in patients with each type of cancer who underwent various therapeutic modalities including RT. The primary emphasis of the search for research articles was on RT as a treatment modality. However, the majority of cancer patients are treated using a combination of various approaches. Our aim in this review was to identify and include studies or articles from which important findings were reported and recently published.

### Brain malignancies

Among brain tumors, primary radical resection is the most essential part of treatment, and studies have also included patients who underwent primary surgery and postoperative RT (PORT) in their treatment sequence. Yersal et al. [37] reported negative results for the PLR in glioblastoma patients. Treatment also varied considerably, and progression-free survival (PFS) and overall survival (OS) did not significantly differ according to the PLR. Gao et al. [38] analyzed 274 atypical meningioma patients. The preoperative PLR was significantly associated with PFS according to the receiver operating characteristic curve. The PLR, included in the risk model, was also significantly correlated with PFS in multivariate analysis. Hsu et al. [39] analyzed the results of 182 malignant glioma patients. A Post-RT PLR > 200 but not an intra-RT PLR > 200 was significantly associated with improved OS and PFS in multivariate analysis. A summary of the studies is shown in Table 1.

**Table 1:** PLR studies in brain malignancies

Year	Article	Total *N*	Study end-point(s)	Disease(s)	Treatment(s)	Important results
2018	Yersal et al. [37]	104	PFS, OS	Glioblastoma	S-> CRT/RT/CTx/No Tx	PFS and OS did not differ significantly according to PLR values
2021	Gao et al. [38]	274	PFS	Atypical Meningioma	S-> RT	Preop PLR was significantly associated with PFS (ROC analysis); PLR including risk score significantly correlated with PFS in multivariate analysis
2022	Hsu et al. [39]	182	OS, PFS	Malignant gliomas	S-> RT	Post-RT PLR > 200 was significantly associated with improved OS and PFS in multivariate analysis

PFS: Progression free survival; OS: Overall survival; S: Surgery; CRT: Chemoradiation therapy; RT: Radiation therapy; CTx: Chemotherapy; Tx: Treatment; PLR: Platelet to lymphocyte ratio; ROC: Receiver Operating Characteristic

### Breast malignancies

In an early study by Krenn-Pilko et al. [40], 793 nonmetastatic breast cancer patients were analyzed. In accordance with traditional treatment guidelines, most of the included patients underwent breast-conserving surgery and adjuvant RT. An increased preoperative PLR was significantly associated with decreased cause-specific survival (CSS) and OS in multivariate analysis. An increased PLR was significantly associated with the occurrence of distant metastases (DM) in univariate analysis. Although treatment methods and disease stages were heterogeneous because of the meta-analysis feature, a high PLR was associated with poor disease-free survival (DFS) and OS in the study by Zhang et al. [41]. A greater incidence of high PLR was noted in the stage II–IV subgroup than in the stage I subgroup. In addition, the incidence of high PLR was significantly different between the lymph node-positive and lymph node-negative groups and between the metastasis-positive and metastasis-negative groups. Although treatment types were not specified in detail, a lower PLR (≤ 210) correlated with a better DFS among patients with inflammatory breast cancer [29]. The results of these studies on breast cancer are summarized in Table 2.

**Table 2:** PLR studies in breast malignancies

Year	Article	Total *N*	Study end-point(s)	Disease(s)	Treatment(s)	Important results
2014	Krenn-Pilko et al. [40]	793	CSS, OS, DMFS	Breast ca.	BCS + RT (90%), MRM (10%)	The elevated preoperative PLR was significantly associated with CSS and OS in multivariate analysis; increased PLR showed a significant association with the occurrence of distant metastases in univariate analysis
2017	Zhang et al. [41]	5542 (12 studies)	DFS, OS	Breast ca.	Not specified	High PLR was associated with poor DFS and OS; higher incidence of high levels of PLR was noted in the stage II–IV group relative to the stage I group; the incidence of high levels of PLR was significantly different between the lymph node-positive and lymph node-negative groups and between the metastasis-positive and metastasis-negative group
2020	Van Berckelaer et al. [29]	127	RFS, DMFS, OS	Inflammatory breast ca.	Not specified except NACT	A lower PLR (≤ 210) was correlated with better RFS and DMFS; a high PLR was significantly associated with metastatic disease in multivariate analysis

CSS: Cause specific survival; OS: Overall survival; DMFS: Distant metastasis free survival; BCS: Breast conserving surgery; RT: Radiation therapy; MRM: Modified radical mastectomy; PLR: Platelet to lymphocyte ratio; DFS: Disease free survival; RFS: Relapse free survival; NACT: Neoadjuvant chemotherapy; DMFS: Distant metastasis free survival

### Gastrointestinal malignancies

The PLR has been widely studied in various gastrointestinal (GI) malignancies. Like in other studies, an increased PLR was associated with inferior outcomes in most of these studies. Several types of study endpoints demonstrated a close relationship with the PLR. The major results of GI malignancy studies are summarized in Table 3.

**Table 3:** PLR studies in gastrointestinal malignancies

Year	Article	Total *N*	Study end-point(s)	Disease(s)	Treatment(s)	Important results
2017	McLaren et al. [28]	60	pCR, OS	Esophageal ca.	nCRT-> S	Increased pretreatment PLR was the predictor of poor pCR; only pretreatment PLR was predictive of decreased OS in Cox model
2021	Khin et al. [44]	53	OS, PFS	Esophageal ca.	dCRT	PLR significantly increased after CRT; higher levels of PLR before and after CRT were associated with inferior OS; post-CRT PLR ≥ 420 had inferior OS and PFS compared to PLR < 420 in univariate analysis; greater increase in PLR (∆PLR ≥ 230) after CRT was associated with inferior OS and PFS in univariate analysis
2021	Wang et el [42].	113	OS, PFS	Esophageal ca.	S only/CRT	Pretreatment PLR was significantly correlated with LN and distant organ metastasis; pretreatment PLR (> 183.06) was independently associated with poor OS or PFS in multivariate analysis
2022	Ran et al. [47]	80	OS, PFS	Esophageal ca.	dCRT	Pretreatment dichotomized PLR was not significantly correlated with OS or PFS
2022	Zhang et al. [45]	106	OS, DFS	Esophageal ca.	dCRT	The change of PLR (ΔPLR) was the independent prognostic factors in OS and DFS; higher ΔPLR was related to poor OS and DFS; high ΔPLR was associated with radiation pneumonitis
2022	Du et al. [46]	245	OS, PFS	Esophageal ca.	dCRT	Pretreatment PLR > 148 was associated with inferior OS in only univariate analysis
2022	Tseng et al. [43]	420	OS, DSS	Esophageal ca.	dCRT	Low levels of pretreatment PLR (< 375) was independently associated with better OS and DSS; elevated PLR after treatment had better DSS in univariate analysis
2023	Sun et al. [48]	353	cTR	Esophageal ca.	dCRT	High PLR was independently associated with worse RT tumor response
2021	Ajdari et al. [49]	89	LF, OS	HCC	SBRT for liver metastasis	Baseline PLR significantly associated with OS
2022	Li et al. [50]	309	OS	HCC	IMRT for unresectable HCC/TACE before IMRT (66%), resection (48.2%)	PLR significantly increased after IMRT; pre-PLR, post-PLR and delta-PLR were significantly associated with OS in univariate analysis
2022	Park et al. [51]	39	LC, OS, PFS	HCC	SBRT	PLR increased after SBRT, and decreased slowly to the pre-SBRT value at 6-months; the PLR change was significantly associated with OS in multivariate analysis; post-SBRT PLR > 90 was associated with poorer OS and PFS in univariate analysis
2022	Bae et al. [31]	4076	IHRFS, OS	HCC	LT/S/RFA/TACE + RFA/TACE + RT/RT	Pretreatment PLR and post-treatment worsening of PLR was independently associated with IHRFS; post-treatment worsening of PLR was significantly related to both early and late IHR
2023	Lee et al. [12]	76	OS, DC, LC, IHC	HCC	TACE/RT, RT	The highest post-treatment PLR was the independent prognostic indicator of DC rates; higher post-treatment PLR (> 235.7) was associated with poor DC rates
2018	Lee et al. [55]	297	pCR	Rectal ca.	nCRT-> S	PLR during CRT and change of PLR during CRT were the independent predictors of pCR in multivariate logistic regression
2021	Partl et al. [57]	363	Sphincter preserving surgery	Rectal ca.	nCRT-> S	PLR was not significantly associated with the type of surgery
2022	Miyakita et al. [53]	168	Lateral LN recurrence	Rectal ca.	nCRT-> S	High pre- and post-CRT PLR were significantly associated with lateral LN recurrence
2022	Ergen et al. [52]	53	pTR, DFS, OS	Rectal ca.	nCRT-> S	PLR was the significant prognostic factor for OS and DFS in ROC analysis; high PLR was significantly associated with poor OS and DFS in multivariate analysis
2022	Duque-Santana et al. [26]	100	pCR, DFS, OS	Rectal ca.	nCRT-> S	High pretreatment PLR (> 133) was the significant predictor of inferior DFS in multivariate analysis
2022	Huang et al. [33]	69	OS, DFS	Rectal ca.	nCRT-> S	The median OS was significantly higher among patients with a high post-CRT PLR than among those with a low post-CRT PLR
2022	Xu et al. [56]	205	pTR, OS, DFS	Rectal ca.	nCRT-> S	After propensity score matching, good response group displayed significantly lower pre-CRT PLR; there were no significant difference of post-CRT PLR according to response groups
2022	Chiloiro et al. [54]	808	pCR, OS, DFS	Rectal ca.	nCRT-> S	Pretreatment PLR was not associated with pCR, OS or DFS

pCR: Pathological complete response; OS: Overall survival; nCRT: Neoadjuvant chemoradiation therapy; S: Surgery; PLR: Platelet to lymphocyte ratio; PFS: Progression free survival; dCRT: Definitive chemoradiation therapy; LN: Lymph node; DFS: Disease free survival; DSS: Disease specific survival; cTR: Clinical tumor response; RT: Radiation therapy; LF: Local failure; HCC: Hepatocellular carcinoma; SBRT: Stereotactic body radiation therapy; IMRT: Intensity modulated radiation therapy; TACE: Transarterial chemoembolization; LC: Local control; IHRFS: Intrahepatic relapse free survival; LT: Liver transplantation; S: Surgery; RFA: Radiofrequency ablation; DC: Distant control; IHC: Intrahepatic control; pCR: Pathological complete response; nCRT: Neoadjuvant chemoradiation therapy; LN: Lymph node; pTR: Pathological tumor response; DFS: Disease free survival; ROC: Receiver operating characteristic

In esophageal cancer, pathological tumor response, DFS, PFS and OS were the main study endpoints. The pretreatment and post-treatment PLR or the change in PLR was investigated. The pretreatment PLR was significantly correlated with lymph node (LN) and distant organ metastasis according to Wang et al. [42]. In contrast to most related studies, Tseng et al. [43] reported that an elevated PLR after treatment was associated with better disease-specific survival (DSS). Changes in the PLR have also shown prognostic significance in several studies [43–45]. A greater change in the PLR was associated with inferior DFS and OS in the studies by Khin et al. [44] and Zhang et al. [45], whereas an elevated PLR after treatment was associated with better DSS in the study by Tseng et al. [43]. Results indicating no correlation or statistical significance in only univariate analysis between PLR and study endpoints (OS and DFS) were also reported [46, 47]. Correlation between high PLR and worse pathological or clinical tumor response was also illustrated [28, 48].

In hepatocellular carcinoma (HCC), the baseline PLR (pretreatment), post-treatment PLR and change in PLR were also correlated with OS and intrahepatic relapse-free survival [12, 31, 49–51]. Lee et al. [12] reported that the highest post-treatment PLR was an independent prognostic indicator of distant control rates among patients who underwent curative intent trans-arterial chemoembolization followed by fractionated or stereotactic ablative RT. Post-treatment worsening (increase) of the PLR was significantly related to intrahepatic recurrence in Bae et al. [31]. The results of other studies on HCC are described in Table 3.

In rectal cancer, both positive and negative results have been described. In positive studies, a high PLR was significantly associated with worse outcomes with decreased OS and DFS [26, 52]. Lateral LN recurrence was also correlated with high PLR in the study by Miyakita et al. [53]. The relationship between tumor response and the PLR was also studied [54–56]. In studies with negative results, the PLR was not associated with OS, DFS or tumor response [54, 57]. The opposite results were also reported in relation to other studies, in which a high PLR was associated with a better prognosis (better OS) [33].

### Genitourinary and gynecological malignancies

PLR studies also demonstrated similar results as other site malignancies in genitourinary and gynecological malignancies. In a large-scale study by Langsenlehner et al. [19], high pretreatment PLR (PLR ≥ 190) was independently associated with poor metastasis-free survival (HR = 2.24), CSS (HR = 3.99), and OS (HR = 1.87) in multivariate analysis among prostate cancer patients who underwent definitive RT. However, in Huszno et al. [58], pretreatment PLR was not associated with OS among prostate cancer cohorts comprised with patients who underwent RT or surgery. Cervix cancer was vigorously studied for PLR investigation in gynecological cancer. Meta-analysis by Ma et al. [34] has shown that elevated pretreatment PLR was significantly correlated with poor major study outcomes such as OS, DFS or PFS. The PLR was also meaningfully correlated with lymphovascular invasion, LN metastasis, large tumor size (> 4 cm) and high grade (G3) tumors. The results were statistically significant although the study population was mixed. Clinical response was also another significant end-point in Chauhan et al. [59] and high pretreatment PLR significantly associated with poor response. Statistically significant and independent inferior OS or PFS was reported resulting from high pretreatment PLR in other literatures [27, 60]. The major study results of genitourinary and gynecological malignancies are summarized in Table 4.

**Table 4:** PLR studies in genitourinary and gynecological malignancies

Year	Article	Total *N*	Study end-point(s)	Disease(s)	Treatment(s)	Important results
2015	Langsenlehner et al. [19]	374	MFS, CSS, OS, Biochemical DFS	Prostate ca.	RT	High pretreatment PLR (PLR ≥ 190) was associated with poor MFS, CSS and OS in multivariate analysis
2022	Huszno et al. [58]	152	OS	Prostate ca.	RT (81.6%)/S (13.8%)	Pretreatment PLR was not associated with OS
2018	Ma et al. [34]	3668 (12 studies)	OS, DFS, PFS	Cervix ca.	CRT, mixed, surgery	Elevated pretreatment PLR was significantly correlated with poor OS, DFS/PFS; elevated pretreatment PLR was highly correlated with lymphovascular invasion (+), lymph node metastasis (+), tumor size (> 4 cm), and grade (G3)
2022	Chauhan et al. [59]	90	Clinical response	Cervix ca.	CRT	High pretreatment PLR was significantly associated with poor response (AUC = 0.626)
2023	Gao et al. [27]	110	OS, PFS	Cervix ca.	RT	High pretreatment PLR (PLR > 187.88) was the independent risk factor for inferior OS; high PLR was associated with LN metastasis
2023	Li et al. [60]	795	OS	Cervix ca.	CRT	High pretreatment PLR (> 164.29) was independently associated with inferior OS

MFS: Metastasis free survival; CSS: Cause specific survival; OS: Overall survival; DFS: Disease free survival; RT: Radiation therapy; PLR: Platelet to lymphocyte ratio; S: Surgery; PFS: Progression free survival; CRT: Chemoradiation therapy; AUC: Area under the curve; LN: Lymph node

### Hematological malignancies

The PLR has not been broadly studied in hematological malignancies. In Wang et al. [61], the PLR was significantly correlated with various lymphoma staging systems and a high PLR was an independent prognostic factor for inferior OS in patients with extranodal NK/T-cell lymphoma. In Hodgkin and mucosa-associated lymphoid tissue (MALT) lymphoma, a high PLR was independently linked to worse outcomes [62, 63]. The study results for patients with hematological malignancies are summarized in Table 5.

**Table 5:** PLR studies in hematological malignancies

Year	Article	Total *N*	Study end-point(s)	Disease(s)	Treatment(s)	Important results
2014	Wang et al. [61]	252	OS	Extranodal NK/T-cell lymphoma, nasal type	CRT/CTx/RT/No Tx	PLR significantly correlated with AAS, IPI, KPI and ALC; high PLR (> 185) was the independent prognostic factor of inferior OS, and PLR including prognostic model significantly predicted OS
2018	Reddy et al. [62]	338	FFP	Hodgkin lymphoma (classic type)	Unspecified	High pretreatment PLR was the independent prognostic factor of FFP in multivariate analysis
2023	Wen et al. [63]	183	PFS	MALT lymphoma	At least 1 antitumor therapy (S/RT/CTx /TT/Anti-HP therapy)	High pretreatment PLR (> 131.47) was the independent prognostic factor of PFS; PLR-based nomogram significantly predicted PFS

OS: Overall survival; NK: Natural killer; CRT: Chemoradiation therapy; CTx: Chemotherapy; RT: Radiation therapy; Tx: Treatment; PLR: Platelet to lymphocyte ratio; AAS: Ann arbor stage; IPI: International prognostic index; KPI: Korean prognostic index; ALC: Absolute lymphocyte count; FFP: Freedom from progression; PFS: Progression free survival; MALT: Mucosa-associated lymphoid tissue; S: Surgery; TT: Targeted therapy; HP: Helicobacter pylori

### Head and neck malignancies

The PLR was widely investigated in various types of head and neck malignancies. Nasopharyngeal, oropharyngeal and hypopharyngeal cancers were routinely treated with definitive RT and chemotherapy as known treatment guidelines. Upfront surgery was preceded in oral and salivary gland cancers. In meta-analysis published in 2020, 3459 patients were included among 9 nasopharyngeal cancer studies [30]. An increased pretreatment PLR predicted poor OS, PFS and DM free-survival (DMFS) in non-metastatic disease, whereas an increased PLR was not significantly associated with poor OS in patients with metastatic disease. A high PLR was significantly related to worse OS, PFS or DMFS in majority of the studies [30, 35, 64–67]. However, opposite [68] and negative results [69, 70] have also been reported. In 418 patients with salivary gland cancer by Yan et al. [67], a high PLR before PORT was significantly associated with worse DMFS in multivariate analysis, and PLR-based nomogram also conveyed accurate individual predictions of DMFS. In oral squamous cell cancers, a high preoperative PLR was associated with inferior DFS or OS [65, 71]. The summarized results are described in Table 6.

**Table 6:** PLR studies in head and neck malignancies

Year	Article	Total *N*	Study end-point(s)	Disease(s)	Treatment(s)	Important results
2018	Jiang et al. [69]	247	NPC	OS, PFS, DMFS, LRFS	(C)RT	Pretreatment PLR was significantly associated with T-stage and tumor stage; pretreatment PLR was not significantly associated with OS, PFS, DMFS and LRFS
2020	Tazeen et al. [71]	130	Oral ca.	DFS, OS	S, S-> RT/CTx/CRT	Preoperative high PLR (> 142) was the independent factor for DFS and OS
2020	Zhang et al. [30]	3459 (9 studies)	NPC	OS, PFS, DMFS	(C)RT	Increased pretreatment PLR predicted poor OS, PFS and DMFS in non-metastatic disease; increased PLR was not significantly associated with poor OS in patients with metastatic disease
2021	Chen et al. [68]	216	NPC	OS, RRFS, LRRFS, DMFS	(C)RT	Low pre-treatment PLR (≤ 140.065) remained significantly related to worse OS and DMFS
2021	Li et al. [64]	342	NPC	OS, PFS	(C)RT	High PLR (≥ 184.91) was significantly associated with poor OS and PFS
2021	Peng et al. [35]	1661	NPC	OS, PFS	(C)RT, CTx-> CRT, CRT-> CTx	High pretreatment PLR (> 157.14) was significantly associated with inferior OS and PFS; PLR-including risk score significantly predicted OS and PFS
2021	Staniewska et al. [70]	208	OPC	OS	(C)RT, CTx-> RT	Pretreatment PLR was not associated with OS
2022	Wan et al. [66]	103	HPC	OS, PFS	(C)RT, CTx-> RT	After PSM, high pretreatment PLR (≥ 133.06) was significantly associated with inferior OS, PFS and LF
2023	Yan et al. [67]	418	SGC	DMFS	S-> RT	Pre-PORT high PLR was significantly associated with worse DMFS in multivariate analysis; PLR-based nomogram presented accurate individual prediction of DMFS
2023	Liu et al. [65]	418	Oral ca.	OS	S-> RT and/or CTx	High preoperative PLR (> 135) was associated with inferior OS

NPC: Nasopharyngeal cancer; OS: Overall survival; PFS: Progression free survival; DMFS: Distant metastasis free survival; LRFS: Local relapse free survival; CRT: Chemoradiation therapy; PLR: Platelet to lymphocyte ratio; DFS: Disease free survival; S: Surgery; RT: Radiation therapy; CTx: Chemotherapy; RRFS: Regional recurrence free survival; LRRFS: Locoregional recurrence free survival; OPC: Oropharyngeal cancer; HPC: Hypopharyngeal cancer; PSM: Propensity score matching; LF: Local failure; SGC: Salivary gland cancer; PORT: Postoperative radiation therapy

### Lung malignancies

The PLR has also been extensively investigated in small cell lung cancer (SCLC) and non-small cell lung cancer (NSCLC). In SCLC, a high PLR was associated with inferior OS or PFS in several studies [36, 72, 73], while no correlation was also reported [25, 74]. In NSCLC, a variety kind of study cohorts were enrolled in the studies. Pre- or mid-treatment PLR was correlated with OS or PFS [75, 76]. In early-stage NSCLC, low pretreatment PLR was significantly associated with superior non-local failure following stereotactic body radiation therapy (SBRT) [77]. Delikgoz Soykut et al. [75] described interaction between the PLR and loco-regional relapse free survival. Negative results with no correlation between the PLR and clinical outcomes were also depicted [25, 78]. Pavan et al. [79] explored tumor immune-milieu among patients with superior sulcus NSCLC who underwent conformal RT followed by surgery. CD68 + tumor infiltrating immune cells were associated with a higher PLR and higher PLR values seemed to be linked with fewer residual viable tumor cells. However, the presurgical PLR was not associated with a radiological or metabolic response. The results are described in Table 7.

**Table 7:** PLR studies in lung malignancies

Year	Article	Total *N*	Study end-point(s)	Disease(s)	Treatment(s)	Important results
2015	Cannon et al. [77]	149	OS, Nonlocal failure	ES-NSCLC	SBRT	Low pretreatment PLR (< 250) was significantly associated with superior nonlocal failure in multivariate analysis
2019	Suzuki et al. [72]	122	OS	LS-SCLC	(C)RT	High pretreatment PLR (≥ 140.1) was associated with inferior OS in multivariate analysis
2019	Zhang et al. [36]	286	OS, PFS	LS-SCLC	CTx, (C)RT, CTx-> RT, Surgery	High pretreatment PLR (> 152.1) was associated with inferior OS and PFS in multivariate analysis
2020	Xia et al. [76]	244	OS, PFS	LA-NSCLC	(C)RT	PLR 1-month after initiation was significantly associated with OS and PFS; 1-month PLR was associated with baseline count and mean body dose in multivariate analysis
2020	Yu et al. [73]	544	OS	LS-SCLC	CRT	Pretreatment PLR (continuous variable) was significantly correlated with OS in multivariate analysis; PLR-including model more accurately predicted OS than conventional model
2021	Qi et al. [74]	344	OS	LS-SCLC	CRT, CTx-> CRT	Pretreatment PLR was not significantly associated with OS
2022	Delikgoz Soykut et al. [75]	392	OS, PFS, LRRFS	LA-NSCLC	CRT	Low (< 166) pretreatment PLR was significantly associated with improved OS, PFS and LRRFS in multivariate analysis
2022	Abravan et al. [25]	2513	OS	NSCLC, SCLC	fRT, SBRT	Pretreatment PLR was not significantly associated with OS
2022	Pavan et al. [79]	8	TIIC, PD-L1-TPS, RVTC	SS-NSCLC	CRT-> S	CD68+-TIICs were associated with higher PLR; presurgery PLR was not linked to radiologic or metabolic response; higher PLR values seemed linked with lower RVTC
2022	Aduquaye et al. [78]	72	OS, RFS	ES-NSCLC	SBRT	Pretreatment PLR was not associated with RFS or OS in multivariate analysis

OS: Overall survival; ES-NSCLC: Early stage non-small cell lung cancer; SBRT: Stereotactic body radiation therapy; PLR: Platelet to lymphocyte ratio; LS-SCLC: Limited stage small cell lung cancer; CRT: Chemoradiation therapy; PFS: Progression free survival; CTx: Chemotherapy; RT: Radiation therapy; LA: Locally advanced; LRRFS: Locoregional relapse free survival; fRT: Fractionated radiation therapy; TIIC: Tumor immune infiltrating cell; PD-L1: Programmed death ligand 1;TPS: Tumor proportion score; RVTC: Residual viable tumor cells; SS: Superior sulcus; RFS: Relapse free survival

### Soft tissue malignancies

The PLR was not studied well in soft tissue sarcomas. In the study by Fiore et al. [32], 423 patients with retroperitoneal sarcoma were examined who underwent preoperative RT or RT and chemotherapy. Prognostic index comprised with initial (pretreatment) PLR significantly discriminated OS and served as an available prognostic tool in that study. Tepper et al. [80] reported treatment outcomes of 86 patients with undifferentiated pleomorphic sarcoma (UPS). All included patients underwent resection of the UPS and RT was performed via by neoadjuvant or adjuvant sequence. A high pretreatment PLR was associated with worse OS in univariate analysis but not in multivariate analysis.

### Toxicities

Notably, significant correlation between the PLR and treatment toxicity has been illustrated in several studies. Among 379 esophageal cancer patients who underwent chemoradiation treatment (CRT), Han et al. [81] reported that a high pretreatment PLR was an independent predictor of esophageal fistula, and a nomogram including the PLR significantly predicted esophageal fistula. Yang et al. [82] reported that an increased PLR during 3–4 weeks of RT was the independent indicator of symptomatic radiation pneumonitis among patients with esophageal cancer who underwent RT or CRT, and PLR-based nomogram also significantly predicted symptomatic radiation pneumonitis. In contrast, an increased 6-week PLR was associated with a decreased risk of radiation pneumonitis among patients with NSCLC in Huang et al. [83]. Radiation esophagitis was investigated by Qui et al. [84]. A high pretreatment PLR was the significant predictor of severe esophagitis among SCLC patients. Table 8 provides a summary of studies.

**Table 8:** PLR studies related to treatment toxicities

Year	Article	Total *N*	Study end-point(s)	Disease(s)	Treatment(s)	Important results
2020	Han et al. [81]	379	EF	Esophageal ca.	CRT	High pretreatment PLR (> 153) was the independent factor of EF in multivariate analysis; PLR-based nomogram significantly predicted EF
2021	Yang et al. [82]	174	RP	Esophageal ca.	(C)RT	PLR (≥ 523.78) during 3–4 weeks of RT was the independent predictor of symptomatic RP in multivariate analysis; PLR including nomogram significantly predicted symptomatic RP
2022	Huang et al. [83]	84	OS, PFS, RP	NSCLC	(C)RT/(C)RT + durvalumab	Week 6 PLR ≥ 180 was associated with a lower risk of pneumonitis; week 6 PLR (continuous) was the independent indicator of PFS.
2022	Qui et al. [84]	187	Severe radiation esophagitis (≥ Gr2)	SCLC	(C)RT	Pretreatment high PLR (≥ 231.1) was the predictor of severe RE in univariate analysis only

CRT: Chemoradiation therapy; PLR: Platelet to lymphocyte ratio; EF: Esophageal fistula; RT: Radiation therapy; RP: Radiation pneumonitis; OS: Overall survival; PFS: Progression free survival; NSCLC: Non-small cell lung cancer; Gr: Grade; SCLC: Small cell lung cancer; RE: Radiation esophagitis

## Discussion

**Fig. 1:**
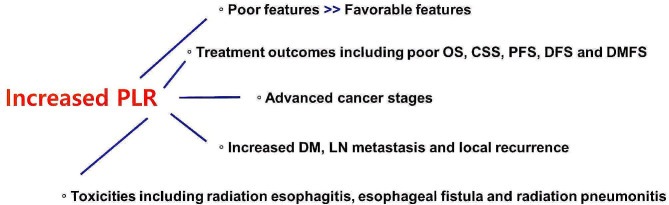
A summary of the clinical implications of the increased PLR

Because of the variety of cancer types, treatment modalities, and timepoints of blood tests, consistent conclusions and results could not be drawn from the review of studies. Although we sought to include, examine and analyze RT-related studies in this review, cancer types treated by RT alone are confined to several diseases only, and most of the malignancies were managed with multidisciplinary and multimodal options. Therefore, we cannot arrive at concrete and constant conclusions from these studies. Multiple blood tests are repeatedly performed even within a short period of time for chronic diseases such as cancer. Accurate quantification of the timing of blood tests seems critical, but there is a possibility of variation among studies. However, despite this heterogeneity, we can focus on several concordant conclusions and results among studies. A summary of the clinical implications of the PLR is illustrated in Fig. 1.

The connection between systemic inflammation and cancer development was described in the 19th century [59]. Since then, the known link between inflammation and cancer has recently undergone a renaissance as a result of investigations of the role of inflammation in cancer [59]. The inflammatory microenvironment is now regarded as the seventh hallmark of cancer according to the numerous clinical and translational studies [85]. Blood parameters such as platelets, leukocytes, lymphocytes, macrophages, monocytes and dendritic cells are regularly measured and compose a paramount part of the immune system. When triggered, these cells release a variety of cytokines and tumor growth-promoting factors. Platelets are among the important tumor-promoting blood cells. They release epidermal growth factor, hepatocyte growth factor, insulin-like growth factor, platelet-derived growth factor, transforming growth factor β, vascular endothelial growth factor and many cytokines that promote epithelial-to-mesenchymal transition and metastasis [86, 87]. Lymphocytes are also an integral part of host’s cellular immunity and are involved in antitumor immune responses. Lymphocytes can induce cell death and impede the proliferation and migration of cancer cells [33]. The presence of lymphocytes in the tumor is associated with improved treatment responses and favorable prognosis, whereas low lymphocyte counts are linked to diminished antitumor immune responses, which can lead to tumor growth and progression [88].

When we summarized the results, most studies illustrated association between a high PLR and poor outcomes even though consistent conclusions and trends were not found. The majority of the included studies investigated OS, DFS or PFS as primary study endpoints, and a high PLR was correlated with deteriorated outcomes at these endpoints. An association with LC has been observed in few studies, whereas a close relationship with DM has been described in several studies. Connection between a high PLR and worse clinical and pathological factors as well as disease stages have also been demonstrated. The specific relevance of the PLR in particular cancer types can be determined through well-designed studies with fixed and predefined PLR measurement timepoints.

When we examined the results according to disease site, some concordant results were observed despite the heterogeneous composition of the study groups. Among the brain tumors, atypical meningioma and malignant gliomas were included. The preoperative or Post-RT PLR was associated with PFS or OS. The PLR-including risk score also significantly correlated with PFS. In breast cancer, the PLR was investigated from diverse perspectives. In the meta-analysis, the associations of a high PLR with advanced disease stage, LN metastasis, and DM, as well as poor DFS and OS, were reported. Interactions of the preoperative PLR with CSS and OS and a very strong association between a high PLR and DM were also depicted. In GI malignancies, the PLR has been more broadly investigated. In esophageal cancer, most included studies involved patients who underwent definitive CRT. However, studies on the heterogeneity of treatment therapeutics and studies on patients treated with neoadjuvant CRT followed by surgery were also included. Although most of the endpoints were OS, DFS or PFS, tumor response or complete response was another endpoint in some studies. In most of those studies, elevated PLRs were associated with poor clinical outcomes and poor pathological responses. In some studies, the PLR increased after CRT, and a high post-treatment PLR was related to poor OS. Changes in the PLR were also related to OS and DFS in several studies. The assessment timepoints of the PLR in relation to treatment time varied among studies and were linked to treatment outcomes in various aspects. LN metastasis and DM were also linked to a high PLR in some studies. The results for HCC patients were similar to those of other PLR-related studies. The highest PLR among the collected data was associated with DM, and worsening of the PLR after treatment was connected to intrahepatic recurrence. In rectal cancer, according to the standard treatment paradigm, treatment proceeded via the sequence of neoadjuvant CRT followed by radical surgery in all included studies. The results were also divided into positive (close relationship) and negative (no relationship) outcomes. An elevated PLR was related to poor OS, DFS, PFS and lateral LN recurrence. A favorable response after CRT was associated with a low PLR in some studies. In hematologic malignancies, investigations of the PLR have rarely been conducted. Extranodal NK/T-cell lymphoma, Hodgkin’s lymphoma and MALT lymphoma were the disease entities studied. An increased PLR was consistently related to poor OS or PFS in all the included studies. A number of studies on head and neck malignancies have been performed for various types of cancer. In the study population of patients who underwent definitive CRT, an elevated PLR was related to poor OS, PFS or DMFS in most of the studies. Opposite or negative (no relationship) results have been reported from several studies. In the study cohorts of salivary gland cancer or oral cancer, in which upfront surgery was followed by PORT, the PLR before surgery or PORT was connected to DMFS, DFS or OS. Many investigational studies related to the PLR in lung cancer have been carried out in both SCLC and NSCLC. Similar to the finding of other studies, an elevated pretreatment PLR was related to inferior OS and PFS. The relationship with the mid-treatment PLR was also depicted. In one study, a close association with local recurrence was described. PLR-related studies of soft tissue sarcomas have rarely been performed. The pretreatment PLR demonstrated a close relationship with OS. Interestingly, a close connection between the PLR and treatment toxicitiy has been reported in several articles. Esophageal fistula, radiation esophagitis and radiation pneumonitis are representative examples of toxicities that have shown a close relationship with the PLR, and pretreatment or mid-treatment PLR values were significantly linked to toxicities.

## Conclusions

In summary, the PLR was associated with basic tumor characteristics, various oncologic factors, treatment outcomes and toxicities in cancer treatment involving RT. Although the results were not consistent, increased PLR mostly correlated with poor clinical features and prognosis. These features include baseline tumor characteristics; clinical study endpoints such as DFS, DM, DMFS, OS and PFS; and partly LN metastasis or local recurrence. However, some studies have shown negative or opposite results. The consistency of the PLR measurement time points in relation to the treatment and the consistency of treatment types and modalities in the study design should be paramount for drawing more accurate and firm conclusions. Future studies with results that may support more concrete and definitive conclusions are anticipated.

## Data Availability

No datasets were generated or analysed during the current study.

## Abbreviations

RT: Radiation therapy
LMR: Lymphocyte-to-monocyte ratio
NLR: Neutrophil-to-lymphocyte ratio
PLR: Platelet-to- lymphocyte ratio
AAR: Albumin-to-alkaline phosphatase ratio
CTCs: Circulating tumor cells
PFS: Progression-free survival
OS: Overall survival
PORT: Postoperative RT
CSS: Cause-specific survival
DM: Distant metastases
DFS: Disease-free survival
LN: Lymph node
DSS: Disease-specific survival
HCC: Hepatocellular carcinoma
MALT: Mucosa-associated lymphoid tissue
DMFS: DM free-survival
SCLC: Small cell lung cancer
NSCLC: Non-small cell lung cancer
SBRT: Stereotactic body radiation therapy
UPS: Undifferentiated pleomorphic sarcoma
